# Supplementary material for: Close encounters between infants and household members measured through wearable proximity sensors
Source: PLoS One. 2018 Jun 7;13(6):e0198733. doi: 10.1371/journal.pone.0198733 (PMC5991752; doi:10.1371/journal.pone.0198733)
Supplement: S1 Table — (DOCX) [file pone.0198733.s001.docx]

**S1 Table.** **Number of family members, sex and age distribution, time start and time stop of the experiment.**

|  | | **Sex** | | **Age categories** | | | | | **Experimental period** | |
| --- | --- | --- | --- | --- | --- | --- | --- | --- | --- | --- |
| **Households** | **Number of family members** | **F** | **M** | **<6 months** | | **1-5 years** | **20-49**  **years** | **Age >=50** | **Time start** | **Time stop** |
| *Included in data analysis* | | | | | | | | | | |
| H01 | 3 | 2 | 1 | | 1 | 0 | 2 | 0 | 03/03/15 07:00 | 06/03/15 22:00 |
| H02 | 2 | 1 | 1 | | 1 | 0 | 1 | 0 | 24/03/15 07:00 | 28/03/15 22:00 |
| H03 | 4 | 2 | 2 | | 1 | 0 | 2 | 1 | 14/04/15 13:45 | 18/04/15 20:00 |
| H06 | 3 | 1 | 2 | | 1 | 0 | 2 | 0 | 29/10/15 13:40 | 02/11/15 00:00 |
| H07 | 4 | 3 | 1 | | 1 | 0 | 2 | 1 | 03/11/15 11:00 | 06/11/15 12:00 |
| H09 | 4 | 2 | 2 | | 1 | 1 | 2 | 0 | 11/11/15 16:20 | 14/11/15 12:00 |
| H10 | 3 | 2 | 1 | | 1 | 0 | 2 | 0 | 01/12/15 12:00 | 04/12/15 00:00 |
| H11 | 3 | 1 | 2 | | 1 | 0 | 2 | 0 | 01/12/15 12:30 | 05/12/15 23:00 |
| H12 | 6 | 4 | 2 | | 1 | 2 | 2 | 2 | 01/12/15 13:00 | 04/12/15 14:00 |
| H13 | 3 | 2 | 1 | | 1 | 0 | 2 | 0 | 02/12/15 17:50 | 05/12/15 16:15 |
| H14 | 3 | 2 | 1 | | 1 | 0 | 2 | 0 | 17/12/15 14:00 | 20/12/15 23:00 |
| H15 | 4 | 1 | 3 | | 1 | 1 | 2 | 0 | 17/12/15 15:30 | 20/12/15 23:30 |
| H16 | 3 | 1 | 2 | | 1 | 0 | 2 | 0 | 18/12/15 08:45 | 21/12/15 12:30 |
| H17 | 4 | 2 | 2 | | 1 | 1 | 2 | 0 | 21/12/15 18:00 | 24/12/15 19:00 |
| H18 | 3 | 1 | 2 | | 1 | 0 | 2 | 0 | 28/12/15 19:35 | 31/12/15 20:10 |
| H20 | 3 | 1 | 2 | | 1 | 0 | 2 | 0 | 08/01/16 15:00 | 13/01/16 20:00 |
| *Excluded from data analysis* | | | | | | | | | | |
| H02 | 1 | 0 | 1 | | 0 | 0 | 1 | 0 | 24/03/15 07:00 | 28/03/15 22:00 |
| H04 | 4 | 1 | 3 | | 1 | 1 | 2 | 0 | 16/04/15 18:30 | 19/04/15 20:00 |
| H05 | 3 | 2 | 1 | | 1 | 0 | 2 | 0 | 17/04/15 09:00 | 21/04/15 22:00 |
| H08 | 3 | 1 | 2 | | 1 | 0 | 1 | 1 | 03/11/15 15:30 | 04/11/15 18:00 |
| H19 | 5 | 2 | 3 | | 1 | 2 | 2 | 0 | 05/01/16 10:00 | 07/01/16 21:00 |
