# Supplementary material for: Close encounters between infants and household members measured through wearable proximity sensors
Source: PLoS One. 2018 Jun 7;13(6):e0198733. doi: 10.1371/journal.pone.0198733 (PMC5991752; doi:10.1371/journal.pone.0198733)
Supplement: S2 Table — (DOCX) [file pone.0198733.s002.docx]

**S2 Table.** **Network statistics of each household: number of nodes, number of edges, mean degree, network density, and mean daily network density.**

| **Households** | **Number of nodes** | **Number of edges** | **Mean degree** | **Network**  **density** | **Mean daily density** |
| --- | --- | --- | --- | --- | --- |
| H01 | 3 | 3 | 2.0 | 1.0 | 1.0 |
| H02 | 2 | 1 | 1.0 | 1.0 | - |
| H03 | 4 | 6 | 3.0 | 1.0 | 1.0 |
| H06 | 3 | 3 | 2.0 | 1.0 | 1.0 |
| H07 | 4 | 5 | 2.5 | 0.83 | 0.83 |
| H09 | 4 | 5 | 2.5 | 0.83 | 0.83 |
| H10 | 3 | 3 | 2.0 | 1.0 | - |
| H11 | 3 | 3 | 2.0 | 1.0 | 1.0 |
| H12 | 6 | 14 | 4.7 | 0.93 | 0.77 |
| H13 | 3 | 3 | 2.0 | 1.0 | 1.0 |
| H14 | 3 | 3 | 2.0 | 1.0 | 1.0 |
| H15 | 4 | 6 | 3.0 | 1.0 | - |
| H16 | 3 | 3 | 2.0 | 1.0 | 1.0 |
| H17 | 4 | 6 | 3.0 | 1.0 | - |
| H18 | 3 | 3 | 2.0 | 1.0 | 1.0 |
| H20 | 3 | 2 | 1.33 | 0.67 | 0.67 |
