# Supplementary figures and images for: Close encounters between infants and household members measured through wearable proximity sensors
Source: PLoS One. 2018 Jun 7;13(6):e0198733. doi: 10.1371/journal.pone.0198733 (PMC5991752; doi:10.1371/journal.pone.0198733)

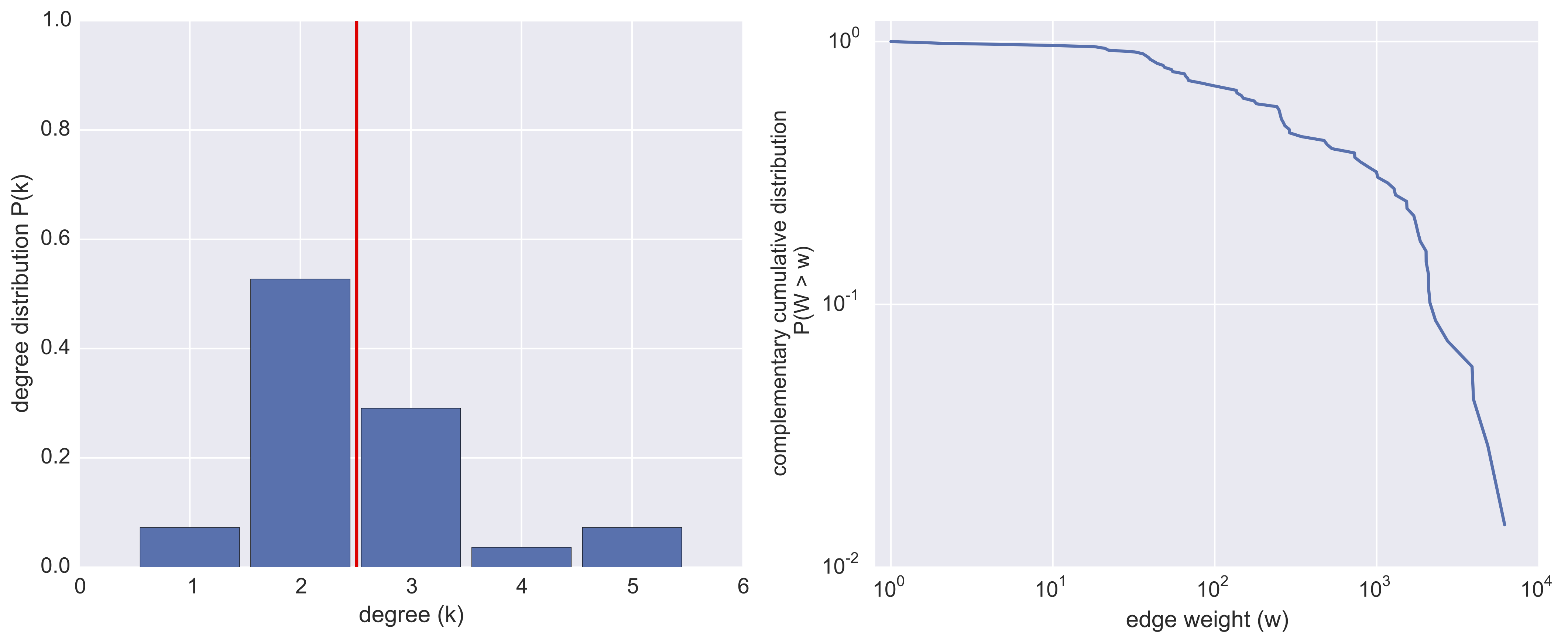

Supplement: S1 Fig — Degree distribution P(k) of the contact network aggregated for all individuals over the whole experimental period, i.e., probability that a randomly chosen node has degree ≥ k. The red line indicates the mean degree value, 〈k〉 = 2.5., and the degree distribution extends between kmin = 1 and kmax = 5 (left panel). Complementary Cumulative Distribution Function (CCDF) of edge weights (i.e. cumulated contact durations) of the aggregated contact network (right panel). (TIFF) [file pone.0198733.s003.tiff]

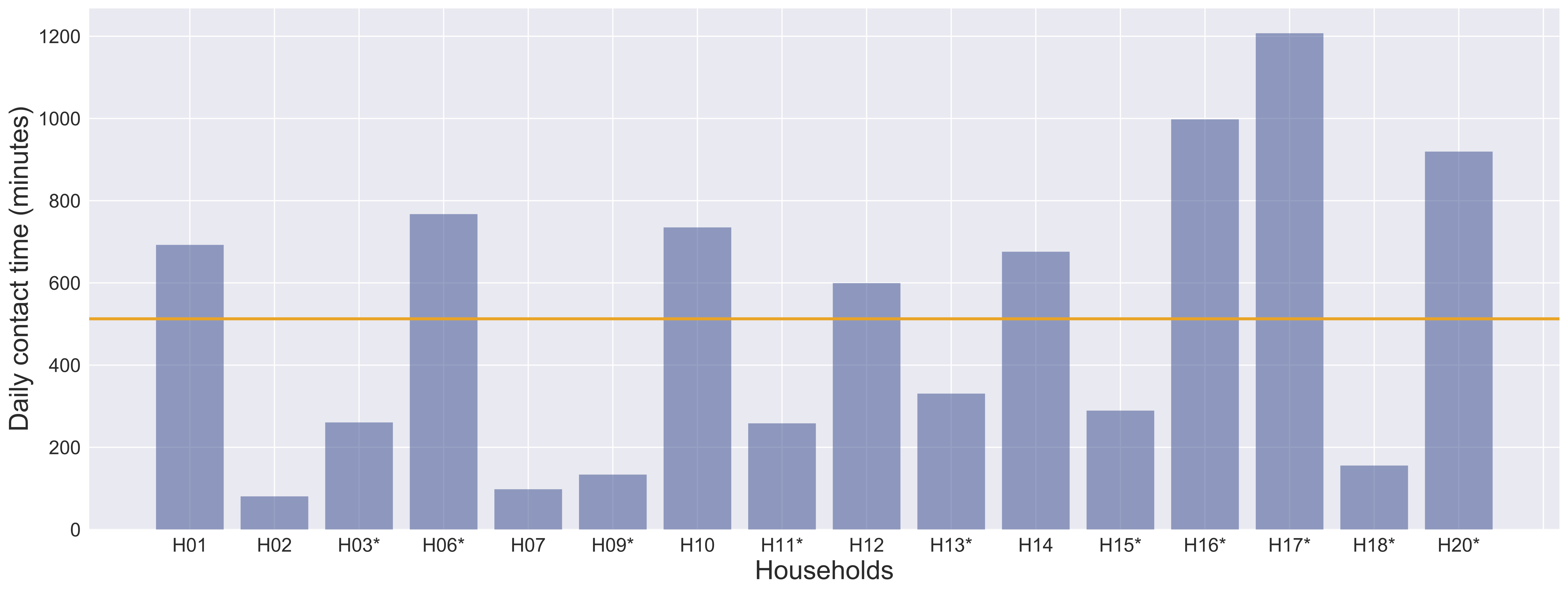

Supplement: S2 Fig — The orange line indicates the mean value (513 minutes). Families in which the infant is exclusively breastfed are labelled with *. (TIFF) [file pone.0198733.s004.tiff]

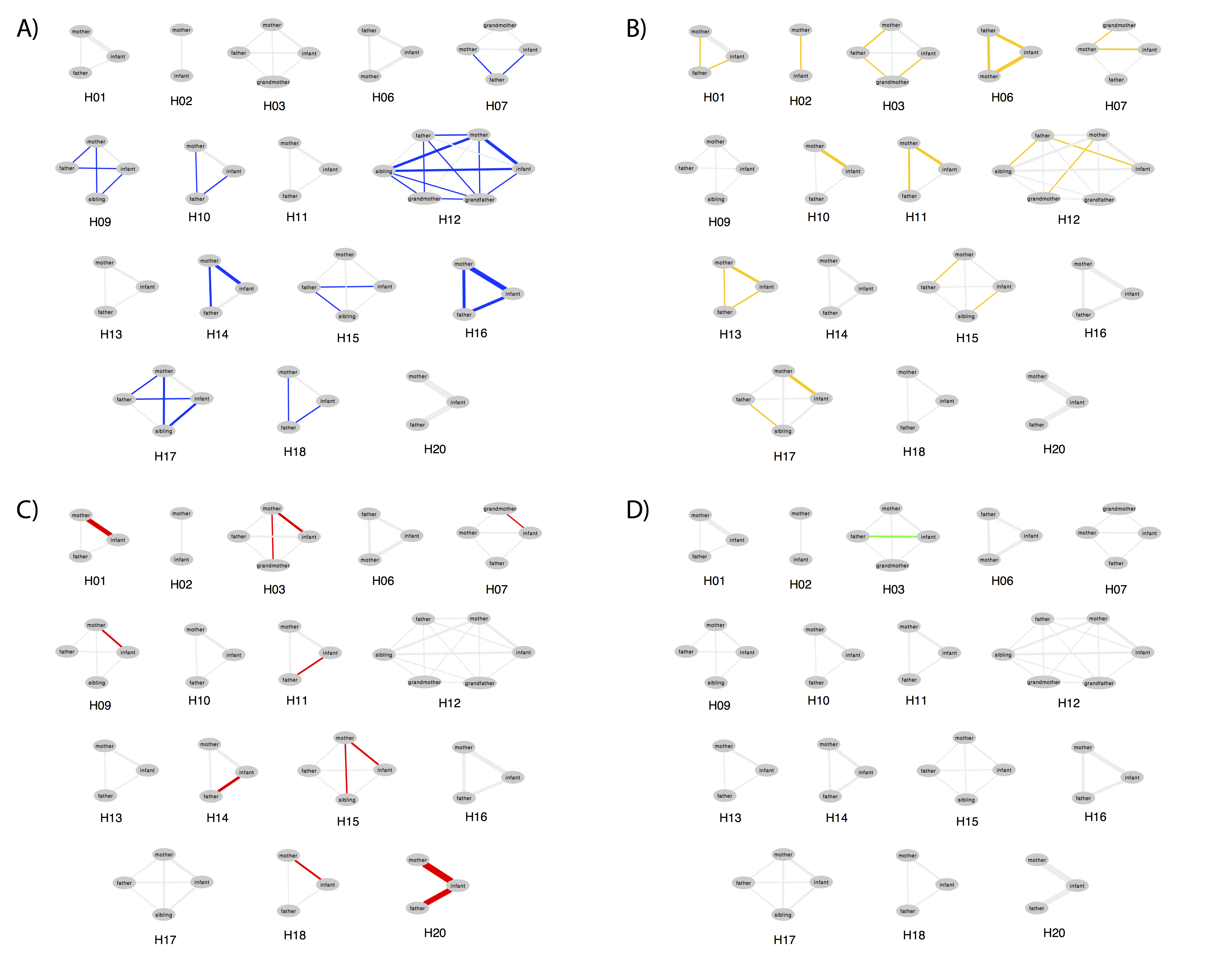

Supplement: S3 Fig — Edge thickness is proportional to the total time spent in proximity by two connected individuals, and the edge colour indicates the time of the day during which it was mainly active: blue = morning (Panel A), yellow = late morning–lunch (Panel B), red = afternoon (Panel C), and green = late afternoon–evening (Panel D). (TIFF) [file pone.0198733.s005.tiff]
